# Supplementary material for: Causal Relationship between Aging and Anorexia Nervosa: A White-Matter-Microstructure-Mediated Mendelian Randomization Analysis
Source: Biomedicines. 2024 Aug 16;12(8):1874. doi: 10.3390/biomedicines12081874 (PMC11351342; doi:10.3390/biomedicines12081874)
Supplement: Supplementary file 1 [file biomedicines-12-01874-s001.zip › Supplementary Figure/Figure S8/Leaveoneout plot of MR analysis for TL to ubm-b-1459.pdf]

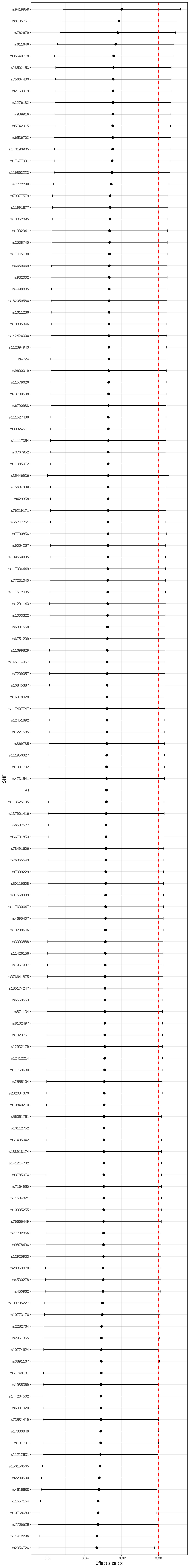
rs9419958
rs8105767
rs762679
rs611646
rs35640778
rs28502153
rs75664430
rs2763979
rs2276182
rs939916
rs5742915
rs6536702
rs143190905
rs17677991
rs116863223
rs7772289
rs79977579
rs11991877
rs13062095
rs1332941
rs2538745
rs17445108
rs6659669
rs932002
rs4498805
rs182059586
rs1611236
rs10805346
rs142426306
rs112394943
rs4724
rs9600019
rs11579626
rs73730598
rs6790988
rs111527438
rs80324517
rs11117354
rs3767952
rs11085072
rs35446936
rs45604339
rs429358
rs76219171
rs55747751
rs7790856
rs6054257
rs139669835
rs117034449
rs77231040
rs117512405
rs1291143
rs1003322
rs6881568
rs6751209
rs11699829
rs145114957
rs7209057
rs10845387
rs16978028
rs117407747
rs12451892
rs7221585
rs869785
rs111950327
rs1907702
rs4731541
All
rs113525195
rs137901416
rs6587577
rs66731853
rs78491606
rs76065543
rs7099229
rs80116508
rs34550383
rs117630647
rs4695407
rs13230646
rs3093888
rs11426156
rs1957937
rs376641875
rs185174247
rs6669563
rs871134
rs8102497
rs1023767
rs12932179
rs12412214
rs11769630
rs2555104
rs202034370
rs10840270
rs56061761
rs10112752
rs61405042
rs188918174
rs141214782
rs3785074
rs7164950
rs11584821
rs10905255
rs76666449
rs77732866
rs9878436
rs12925933
rs28363070
rs4530278
rs450962
rs139795227
rs10773176
rs2282764
rs2967355
rs10774624
rs3891167
rs61748181
rs1985369
rs144204502
rs6007020
rs73581419
rs17803849
rs131797
rs11212631
rs150150565
rs2230590
rs4616688
rs11557154
rs10768683
rs7705526
rs11412296
rs2056726
SNP
−0.06
−0.04
−0.02
0.00
Effect size (b)
